# Is Dysphagia Under Diagnosed or is Normal Swallowing More Variable than We Think? Reported Swallowing Problems in People Aged 18–65 Years

**DOI:** 10.1007/s00455-020-10213-z

**Published:** 2020-11-23

**Authors:** Paula Leslie, David G. Smithard

**Affiliations:** 1 School of Sports & Health Sciences, University of Central Lancashire, Preston, PR1 2HE UK; 2 Geriatric Medicine, Queen Elizabeth Hospital, Lewisham and Greenwich NHS Trust, Stadium Road, Woolwich, London, SE18 4QH UK; 3 University of Greenwich, London, UK

**Keywords:** Dysphagia, Community, Young, Adult, Diagnosis

## Abstract

**Purpose:**

Dysphagia prevalence in younger community dwelling adults and across nations is sparse. We investigated the prevalence of swallowing problems in an unselected cohort of people aged 18–65 years.

**Methods:**

The EAT-10 Assessment Tool was converted into an anonymized online survey. Invitations were e-mailed to author contacts and onwards dispersal encouraged. Analysis was performed using non-parametric test for group comparison (Mann–Whitney *U*) and Spearman’s rho correlation.

**Results:**

From March 2014 to October 2017: 2054 responses (32 reported ages outside of 18–65 or undeclared) from Africa, Asia, Australasia/Oceania, Europe, and North and South America. Responses: 1,648 female, 364 male, (10 reported as both), median age 34, (range 18–65, mean 37.12, SD 12.40) years. Total EAT-10 scores: median 0 (range 0–36, mean 1.57, SD 3.49). EAT-10 score ≥ 3 (337) median 5 (range 3–36, mean 7.02 SD 5.91). Median age 36 (range 19–65, mean 37.81, SD 13.21) years. Declared sex was not statistically significantly associated with non-pathological vs. pathological EAT-10 score (*p* = 0.665). Female scores (median 0.00, mean 1.56, SD 3.338) were significantly higher than for males (median 0.00, mean 1.62, SD 4.161): *U* (Nfemale = 1648, Nmale = 364) = 275,420.000, *z* = − 2.677, *p* = 0.007. Age and EAT-10 score were not associated: females *r*_s_ = − 0.043, *p* = 0.079, *N* = 1648, males *r*_s_ = − 0.003, *p* = 0.952, *N* = 364. Considerable impact on people: “I take ages to eat a main course … This is embarrassing and I often leave food even though I am still hungry.” (no diagnosis, EAT-10 = 17).

**Conclusion:**

Concerns regarding swallowing exist in people undiagnosed with dysphagia, who may feel uncomfortable seeking professional help. Dysphagia may be under reported resulting in a hidden population. Subtle changes are currently seen as subtle markers of COVID-19. Further work is required to ensure that what is an essentially normal swallow does not become medicalized.

## Introduction

Eating and drinking are fundamental to the physical and mental well-being of humankind. Eating and drinking are the essential components of identity and connections across society [1, 2]. Limitations in how we eat and drink have a profound impact on our lives, irrespective of any other physical, psychological, or mental health co-morbidities. Factors affecting eating and drinking are broad ranging and include physical difficulties in managing the food and drink, intolerances, and in the swallow mechanism itself.

Enjoying food and drink requires the ability to swallow safely. Swallowing is a complex multiphase neuro-muscular function, the main purpose of which is to transfer the food or drink (hereafter referred as simply a bolus) safely from the mouth to the stomach. Swallowing is a programmed activity with the primary control being in the brainstem. Cortical input modifies how the bolus moves through the oropharyngeal system depending on the bolus characteristics and number [3]. A safe swallow entails co-ordination between what are typically referred to as the various *phases* of the swallow [4] and the respiratory cycle [5]. Failure for this to happen will result in problems swallowing (dysphagia). Oropharyngeal dysphagia may be a result of problems with neurological control, muscular control, obstruction, or breathing (cardiorespiratory) [6].

Many medical conditions impact the swallow mechanism and hence affect nutrition, hydration, ingestion of medications, as well as the global human aspects associated with eating and drinking. The prevalence of swallowing problems in many medical conditions such as stroke (8–80%), dementia (up to 100%). and Parkinson’s disease (up to 81%) is well documented, although there is still a concern of underreporting [7, 8].

The prevalence of dysphagia in community dwelling older people (> 65 years) has been reviewed over the years. Those studies conducted in the community have used questionnaires such as the EAT-10 designed for those with or without swallowing problems [9] or the Sydney Swallow Questionnaire developed for people with dysphagia and often completed by a nurse or other health care provider if the patient cannot answer [10]. In studies of the elderly where the participants live in the community, the definitions of elderly, the community, and dysphagia vary. This makes comparisons across studies fraught with difficulty; thus; further detail, where required, has been added to allow some comparison between studies (see Table 1) [11–18].

**Table 1:** Studies showing the prevalence of “dysphagia” in community populations

First author, year	Age (years)	Dysphagia definition	*n*	*n* having “dysphagia”
Adkins et al. (2019) [11]	≥ 18	“Difficulty swallowing (food or liquids sticking in your throat or chest, discomfort with swallowing, or choking sensation when swallowing).”	31,129	4998 (16%)
Bhattacharyya (2014) [13] (e-mailed author for detail not in the paper)	Range 18–85 Mean 52.1 (SD 18.9) Median 54	Range of questions such as “DURING THE PAST 12 MONTHS, have you had a SWALLOWING problem such as difficulty eating solid food, taking pills, or drinking beverages?”	34,525	1554 (5%)
Bloem (1990) [12]	Range 87–95 Mean 90 (SD 2.3)	“Are you bothered either by choking or coughing after eating or drinking, or by pieces of food getting stuck in the throat, or swallowing more than once to get the same bite down, or spilling swallowed fluid through the nose?”	130	21 (16%)
Cho (2015) [16]	Mean 63 (SD 16) frequent dysphagia	“In the last year how often had you have difficulty swallowing (a feeling that food sticks in your throat? Frequent = at least weekly Infrequent = less than 1 day a week Either of above”	3669 (3595 used for stats but 74 unaccounted for; thus used 3669 as intention to treat)	168 (5%)
	Mean 62 (SD 15) infrequent dysphagia			937 (26%)
	Mean 61 (SD 16) no dysphagia			1105 (30%)
Eslick (2008) [18] (e-mailed author for detail not in the paper)	Male range 19–100 Mean 46.8 Median 46 Female range 19–75 Mean 45.2 Median 42	“A feeling that food sticks in your throat or chest.”	672	110 (16%)
Kertscher (2015) [14]	Range 18–97 Median 55	EAT-10 cutoff 3 + EAT-10 cutoff 2 +	2,600	219 (8%) 315 (12%)
Nimmons (2016) [15]	Range 50–98 Median (2009) 81 (SD 5)	Sydney Swallow Questionnaire 2009 2012 Feeling of food getting stuck in the throat (Q9) Difficulty swallowing hard foods at present (Q5) A sensation of choking/coughing on swallowing (Q10) Three most common symptoms	634 (of 800)	*(n not reported)* 14%
	Range 50–98 Median (2012) 85 (SD 5)		467 (of 550)	13%
Wilkins et al. (2007) [17]	Minimum age 18 Mean age of those reporting dysphagia 48 (SD 14.4)	Ever having 1 or more episodes of the feeling that food was stuck in their chest or throat or choking or coughing while swallowing?	947	214 (23%)

A further complication is that many studies are based on the gastroesophageal literature, and thus a report of “dysphagia” may be indicating a reflux issue, e.g., when dysphagia is simply defined as “In the last year how often had you have difficulty swallowing (a feeling that food sticks in your throat?)” [16]. Reflux issues may not be assessed at all and are thus difficult to rule out [17]. Both of these situations may lead to high figures that do not necessarily indicate an oropharyngeal dysphagia.

Aside from disease and/or age-related dysphagia, there may be a good proportion of the population with swallowing problems that are hidden from health care support. We need to be careful to not go looking for a problem that does not exist or medicalizing the ends of the range of what is normal. Of merit to consider is that the swallow is a finely tuned process involving several systems and as such problems may show subtle pre-existing conditions or herald the onset of certain diseases before other signs. In pseudo-bulbar type motor neuron disease or tiny lacunar type strokes, a change in the swallow may be the first or only sign due to the need for fine co-ordination of systems. Indeed in the current COVID-19 pandemic, evidence is emerging that subtle signs such as taste and smell may be affected early in the disease [19–24]. Taste and smell are inherent components of the sensory system involved in preparing the gastro-intestinal tract to receive, transport, and process each bolus [25]. More obvious issues are clear when we consider what happens to the interplay between breathing and swallowing in conditions including corona virus respiratory infections.

Even if the difficulties are as a result of lying at the edges of the bell curve of normality, people experiencing problems merit access to further investigation and support. Dysphagia impacts people’s enjoyment of food and drink, their participation in social activities, and the effectiveness of their medications for other conditions. A part of the difficulty with studies in the world of swallowing is that the populations and definitions vary. One study’s “dysphagia” might be another’s more specific aspiration event, similarly the “older” population focus of one might be over 65 and in another over 85 years of age.

## Objective

What is the number of people experiencing eating and drinking problems, and what do they report? Prevalence studies have historically targeted small populations, by locality or access to care. There are no large-scale studies looking at the prevalence of reported swallowing problems across nationalities and continents in the age group 18–65 years. We used the Eating Assessment Tool (EAT-10) to investigate the prevalence of dysphagia in multiple countries and across multiple nationalities. This relatively simple tool is one of the most commonly used in larger sample studies and at the time of the study initiation had the most supporting literature [9].

### Methods

#### Participants

Participation was open to anyone who received the survey and was aged 18–65 years. The survey was distributed through contacts of the authors, via professional lists, student participation (encouraging parents and other family members), and at professional meetings. Wide onward distribution was encouraged. We have no way of knowing the final pool who received the survey, but only the number of people who completed it. No personally identifying information was collected. All data are shared as participants reported them.

#### Ethical Approval

Project (PRO13050556) was reviewed by the University of Pittsburgh Institutional Review Board. Based on the information provided, this project met all the necessary criteria for an exemption and was designated as “exempt” under Section 45 CFR 46.101(b)(2) Tests, surveys, interviews, observations of public behavior on the 18th February 2014.

#### Procedure

The EAT-10 was converted into an online survey using Survey Monkey®. Respondents were invited to score statements on a five-point scale from “strongly disagree” to “strongly agree” and to add free comments yielding anonymous data. The survey was sent out to all the contacts known to the authors and continued onward dissemination was encouraged. The survey included demographics questions: age, sex, country of residence, nationality, medications, and previous medical history.

The presence of swallowing problems was analyzed according to Belafsky et al., with a cut-off score for “pathological” ≥ 3 [9]. Recent work has looked at a cutoff of ≥ 2 on the EAT-10 [14], but the literature is limited. We chose to keep our data at the ≥ 3 cutoff to allow comparison with a more sizable body of previous sources.

#### Data Analysis

The data collected are categorical in nature and observational, i.e., a point in time. The data were analyzed using IBM SPSS Statistics for Windows, Version 26.0. software with the Mann–Whitney *U* test for comparison across groups, and Spearman’s rho correlation for non-parametric data [26]. We have analyzed the data using EAT-10 cutoff at ≥ 3 indicating a swallow problem. An a* priori t*wo-tailed alpha level for significance was set at *p* < 0.05.

## Results

### Participant Characteristics and Geographical Location

Over the period from March 2014 to October 2017, we received 2054 completed surveys (of which 32 reported ages outside of 18–65 or undeclared) resulting in a study pool of 2022 participant responses. Declared sex of the participants was 1648 reported as female (364 reported as male, 10 reported as both).

Reported scores from the EAT-10 across the whole sample of 2022 participants, and the subgroup scoring ≥ 3 (*n* = 337, 17% of total sample), are shown in Table 2. Of these 337 responses, 166 selected n/a to the existing medical conditions where the options were head injury, stroke, Parkinson’s disease, motor neuron disease, head and neck cancer, heartburn, other (please specify free text), or n/a (not applicable).

**Table 2:** EAT-10 scores for whole sample and those scoring ≥ 3

	Sample	EAT-10 ≥ 3
N*	2022	337
Median EAT-10 scores	0	5
Mode EAT-10 scores	0	3
Range EAT-10 scores	0–36	3–36
Mean (SD) EAT-10 scores	1.57 (3.49)	7.02 (5.91)
Age (years)	–	–
Median	34	36
Mode	27	22
Range	18–65	19–65
Mean (SD)	37.12 (12.40)	37.81 (13.21)

*N for further results separated by reported sex *excludes* 10 participants who reported both sex options

**Fig. 1:**
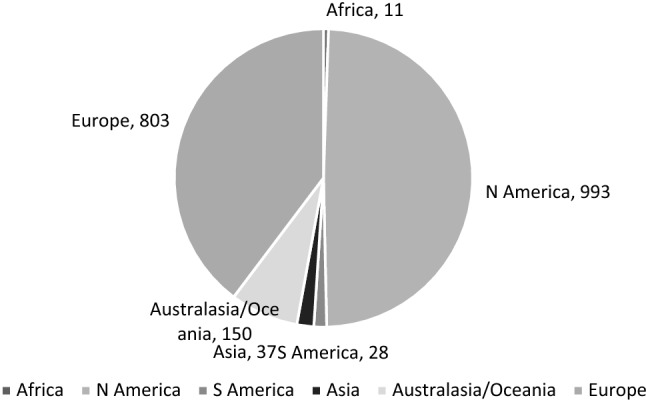
Global responses by continent for all 2022 participants

**Fig. 2:**
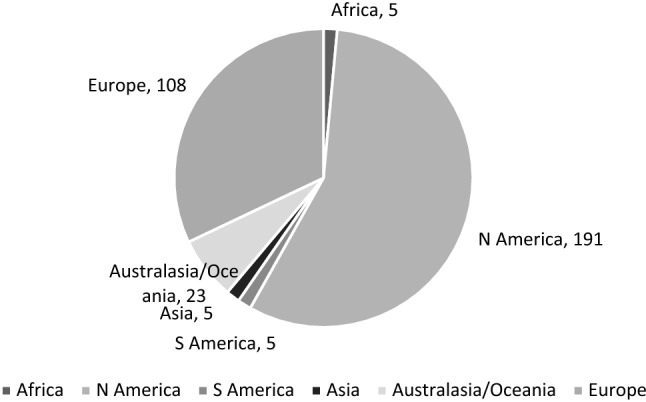
EAT-10 scores ≥ 3 by continent

Responses were received from Africa, North America, South America, Asia, Australia/Oceania, and Europe (see Fig. 1). Responses with scores ≥ 3 are shown in Fig. 2. As a proportion of the total responses from a continent, the “dysphagia” scores were similar with North America at 19% (191/993), Australasia/Oceania at 15% (23/150), and Europe at 13% (108/803). We chose not to report the proportions of the three lowest scoring continents as the numbers were so small.

For the 2012 participants who declared male or female (not both), further analysis was carried out to examine the relation between declared sex and EAT-10 scores (see Table 3). Across the group, female scores: median 0.00, mean 1.56, SD 3.338, and male scores: median 0.00, mean 1.62, SD 4.161. Using a Mann–Whitney *U* test to compare groups, the overall female score was higher than that of the overall male and was statistically significant: *U* (Nfemale = 1648, Nmale = 364) = 275,420.000, *z* = − 2.677, *p* = 0.007. Declared sex was not statistically significantly associated with non-pathological vs. pathological EAT-10 score: *U* (Nfemale = 1648, Nmale = 364) = 297,132.000, *z* = − 0.433, *p* = 0.665.

**Table 3:** Prevalence of EAT-10 ≥ 3 scores by declared sex

Score	0	1	2	≥ 3	Total
Male (*n* = 364)	225	55	26	58	364
Female (*n* = 1648)	865	318	187	278	1648
Total	1090	373	213	336	2012

Spearman’s rho correlation coefficient was used to assess the relationship between age and EAT-10 score. In females, there was no significant correlation between the two *r*_s_ = − 0.043, *p* = 0.079, *N* = 1648. In males, there was no significant correlation between the two *r*_s_ = − 0.003, *p* = 0.952, *N* = 364.

We split our data across decades: 18–30 years, 31–40 years, 41–50 years, and 51–65 years. There is no evidence that there is a physiological difference across these age bands; this was purely to allow comparison with the previous work. Mann–Whitney *U* test was used to examine the relation between declared sex and EAT-10 scores in these age bands and no statistically significant differences were found (see Table 4).

**Table 4:** Age (split into age bands) of those scoring ≥ 3 on EAT-10 by declared sex

Age (years)/EAT-10 by sex	18–30	31–40	41–50	51–65
Male < 3	89	75	62	80
Female < 3	554	334	248	234
Male ≥ 3	13	18	7	20
Female ≥ 3	114	57	44	63
Total	770	484	361	397
Mann–Whitney *U* statistic	39,358.500	14,469.000	7409.000	12,851.000
*z* value	− 1.094	− 1.143	− 1.055	− 0.257
*p* value	0.274	0.253	0.292	0.797

### Participant Comments from Free Text

Several participants commented that they should have been asked if they had a swallow problem at the start of the survey as then they would not have completed it. Interestingly several respondents scored below the cutoff but expressed considerable concern about their swallow as a whole, and/or with features not captured in the survey. The degree to which a person was concerned about their swallow was not necessarily reflected in their EAT-10 score.

Comments from those with a score of 2 or less showed a range of views from no concern, to understanding of why there might be an issue, to great concern:

*I now cough (occasionally choke) 3–4 times per day; usually thin liquids. This is a change from my 50s! (no diagnosis, EAT-10 = 2)*

*Occasionally certain textures e.g. small particules [sic] of apple or rice will enter back of nasal cavity if rushing, not paying attention and not chewing properly. Uncomfortable sensation but does not put me off eating these consistencies. Very fast eater—sometimes have regurgitation immediately post meals. (no diagnosis, EAT-10* = *1).*

*I'll go through periods of choking on things- eg several times a week? due to distractibility or hypersensitivity from the original bout of choking. Otherwise the norm would be choking maybe a few times a year. (no diagnosis, EAT-10* = *1).*

*I have noticed more often in about the past 6 months that I tend to "spontaneously" aspirate saliva (even just when sitting… all of a sudden i'll feel some sneak in the airway and have a coughing jag) (no diagnosis, EAT-10* = *1).*

Of 337 responses with a score or ≥ 3 , there were 166 with no reported illnesses of whom 16 participants reported that they *did not have a swallow problem* in the comments box and shared other information which was categorized into fear (*n* = 3), embarrassment (*n* = 2), eating in public but not mentioning fear/embarrassment (*n* = 3), producing froth, sputum etc. (*n* = 1), were on a feeding tube/TPN (*n* = 2), concern/anxiety (*n* = 1), and coughing who scored 0 on the EAT-10 cough question (4). Comments included:

*Sometimes I will choke on things like onion or lettuce. I’m always the last person to finish eating as I chew a lot to minimize chances of choking or food getting stuck. (no diagnosis EAT-10* = *12).*

*I take ages to eat a main course (30–40 min)—everyone else has finished and I am aware that I am only half way through and I am unable to eat any faster. This is embarrassing and I often leave food even though I am still hungry. I ask for child portions and look for items on the menu that are easy to eat. (no diagnosis EAT-10* = *17).*

*I believe Achalasia is more common than prevalence figures quoted in mainstream literature. My condition was masked by pregnancy and eventually severe weight loss led to premature birth. (late diagnosis EAT-10* = *22).*

## Discussion

This is the largest study to date, covering the widest geographical range, of self-reported swallowing difficulties in community populations. The proportion of people in North America (19%), Australasia/Oceania (15%), and Europe (13%) who reported an EAT-10 score high enough to be classed as pathological is in line with the previous studies (see Table 1).

We did not set out to address the health care seeking behaviors of participants but did receive reports that people were embarrassed to approach health care providers. We know from previous research that a large proportion of people typically do not seek help despite a experiencing a significant impact on physical and psychological well-being. Wilkins et al. found 2% of 947 people ≥ 18 years of age reported dysphagia at least several times a month and 46% had not reported the problem to their general practitioner [17]. Adkins et al. reported 2445 of 4998 (49%) survey participants had not consulted a health care practitioner, and of those who did 2449 (96%) had health insurance [11]. In societies where health care is a business, those with financial means (independent wealth or insurance) are more likely to see help earlier and continue to access health care support. This has direct consequences for the impact and treatment of the disease underlying dysphagia.

The degree to which a person was concerned about their swallow was not necessarily reflected in their EAT-10 score. Of interest are the participants who:

1. scored below the cutoff but expressed considerable concern about their swallow, and/or with features not captured in the survey;
2. scored above the cutoff and reported no disease; and
3. scored above the cutoff, reported no disease, and stated in the free text that they had *no swallow problems.*

Group 1 may have clinical issues and the survey tool was not capturing them, or they may not have organic disease but still have concerns. These contrast with the people in Group 2: what is causing their higher scores? This may relate to features of the EAT-10 questions that people report but do not perceive as problematic. It may be due to swallow issues being lower on a list of issues that a person is dealing with.

Group 3 are possibly the most interesting. How can you get such high scores and state that you have no swallow problem? This is not uncommon in clinical practice where you have patients who tell you they *have no swallow problem* but on more subtle questioning they share that they now avoid certain food types, have changed their posture, always take drinks to help move material down their throats, etc. And you may say again “so do you have any problems swallowing?” to which they still say *no*. Our study focused on the age range 18–65 years but similar to many other areas of health people accommodate to changes as they get older presuming them to be a part of the aging process [27]. In common with many other systems in the body, we do not normally see a simple decrease in ability with age and so changes should be investigated.

All three of these groups perhaps represent the disconnect between how professionals view the swallow process and how patients experience and think about it. This mirrors the situation in the world of voice disorders where clinicians and patients disagree, and instrumental and subjective measures do not align [28]. How can this be so we might ask? One aspect is that the two realities (patient experienced and clinician observed) are different, so tools are not measuring the same things. The other aspect is that definitions are so unclear. This is the case in the world of swallowing disorders even among professionals. It is no wonder that patients do not report things that fit our screening and assessment tools particularly when we are looking at complex and subtle systems.

There are people in the community with swallow difficulties who are not receiving support. Dysphagia professionals are aware that the disorder exists where there is an underlying condition. The underlying condition may be insidious and not yet detected, or may be stark such as a stroke but the professionals involved have missed the minor swallow issues in the midst of more weighty problems. There are people who have a condition who do not seek help. This is of particular concern in the current climate where people are avoiding going to hospital for fear of *catching COVID-19* or overburdening already stretched services [29]. A recent study attempted to track patient reported swallow difficulties and general health-related quality of life issues across six months [30]. The authors acknowledged limitations in the work including aspects of the EAT-10 in detecting change in people with mild dysphagia for example, but it was an important step in addressing symptoms from the perspective of the patient rather than the clinical or research professional.

One previous study found a peak in reported dysphagia in the 40–49 year old age band [18]. We did not find evidence of any differences across age bands. This previous result may be an artifact of the questionnaire used: comparison of true numbers but what does *true* mean? The variation in definitions used by authors, shared with participants and others, limits cross study comparison and possibly creates confusion.

### Limitations

Study limitations include use of the EAT-10 itself. At the time the work was being designed we needed a simple tool, suitable for online survey work to reach as large a spread as possible. Recent work has shown that the psychometric properties of the EAT-10 are poor [31–34], thus going forward investigators should consider alternatives. Investigators also need to carefully consider what their definition of dysphagia is, because how this is worded will control the findings.

Further limitations include the inherent volunteer bias that surveys are subject to. The large number of people with low scores shows that the survey was not just taken by those with a vested interest, i.e., those with the condition. The survey is likely to have captured a narrow slice of society: those with access to the internet, and through connections to existing participants.

Respondents to the survey were largely from the USA, Europe, and Australasia/Oceania. Access to, participation in, and perceptions of swallowing difficulties are likely to vary across countries and cultures [35]. Unlike say a broken leg, the swallow process and its relation to the acts of eating and drinking are much less concrete. A person’s perception of their swallow and whether it is problematic is influenced by more than biomechanics. Knowledge of what support is available, how health care professionals tend to react, how issues might influence employment etc., all influence the lens through which a person might engage with services [36].

### Clinical Significance

We need to be careful to not medicalize the ends of the range of normality in the swallow: difference is not necessarily a disorder. Nevertheless, there were a number of respondents who shared the significant impact on their lives. We may need a two pronged approach: partly to raise awareness with the public that swallow difficulties should be checked out, without causing undue concern. Secondarily to educate professionals to ask about how people are managing with eating and drinking. This is difficult in the absence of obvious disease. We in the dysphagia community understand the subtleties and relationship of aspects of the swallow to other areas of health and illness, but not everyone does. Education of the broader health care community is an ongoing process, although promise is being shown by early work such as the 4QT [37]. This tool is designed to be quick and used by any member of a health care team. The 4QT is still in the pilot stage with good sensitivity but poor specificity, which in a screen designed to detect issues for onward assessment is not a bad thing.

We have a current and real concern: rehabilitation in major pandemics where the respiratory system is affected, e.g., COVID-19. The swallow process is inherently interconnected with the respiration systems [38]. Mechanical damage due to intubation or issues of respiratory–swallow co-ordination will need addressing in long-term rehabilitation efforts [39]. People with COVID-19 related and unrelated dysphagia will need care [40]. There is emerging evidence on the pre-COVID-19 conditions that lead to poorer outcomes with an existing baseline of neurological conditions [41] particularly those with pre-existing cerebrovascular disease [42] both of which we know to be high-risk areas for swallow impairments.

## Future Directions

There is enough published literature to consider a systematic review of all the studies focusing on people living in the community. This may be confounded by the range of definitions of what “dysphagia” is. A clear definition of dysphagia or clearly delineated subtypes is required. This would support future researchers to make the many subtly different studies comparable. This would be a worthy aim for the national and international dysphagia research forums. Such a structure would also support the development or modification of existing screening and assessment tools, again to allow for easier comparison.

A formal comparison of clinically assessed and/or instrumentally measured features of the swallow in comparison to what people report with—*and without*—swallow impairments would contribute to the teasing out of what is a swallow problem. In whose eyes does the dysphagia lie: the patient or the professional? Addressing the issue of perception has parallels to, and would contribute to, other areas of health care. These are far more complex issues than the biomechanics of which we know so much. Gaining clarity here might contribute back to definitions and thus guide research to answer the most impactful questions regarding the care of people with swallow difficulties.
